# Quantitative evaluation of an automatic segmentation method for 3D reconstruction of intervertebral scoliotic disks from MR images

**DOI:** 10.1186/1471-2342-12-26

**Published:** 2012-08-02

**Authors:** Chevrefils Claudia, Cheriet Farida, Grimard Guy, Miron Marie-Claude, Aubin Carl-Eric

**Affiliations:** 1 Ecole Polytechnique de Montreal, Biomedical Engineering Institute, Montreal, QC, H3C 3A7, Canada; 2 Sainte-Justine University Hospital Center, Montreal, QC, H3T 1C5, Canada; 3 Department of Computer Engineering and Software, Ecole Polytechnique de Montreal, Montreal, QC, H3C 3A7, Canada; 4 Department of Mechanical Engineering, Ecole Polytechnique de Montreal, Montreal, QC, H3C 3A7, Canada

## Abstract

**Background:**

For some scoliotic patients the spinal instrumentation is inevitable. Among these patients, those with stiff curvature will need thoracoscopic disk resection. The removal of the intervertebral disk with only thoracoscopic images is a tedious and challenging task for the surgeon. With computer aided surgery and 3D visualisation of the interverterbral disk during surgery, surgeons will have access to additional information such as the remaining disk tissue or the distance of surgical tools from critical anatomical structures like the aorta or spinal canal. We hypothesized that automatically extracting 3D information of the intervertebral disk from MR images would aid the surgeons to evaluate the remaining disk and would add a security factor to the patient during thoracoscopic disk resection.

**Methods:**

This paper presents a quantitative evaluation of an automatic segmentation method for 3D reconstruction of intervertebral scoliotic disks from MR images. The automatic segmentation method is based on the watershed technique and morphological operators. The 3D Dice Similarity Coefficient (DSC) is the main statistical metric used to validate the automatically detected preoperative disk volumes. The automatic detections of intervertebral disks of real clinical MR images are compared to manual segmentation done by clinicians.

**Results:**

Results show that depending on the type of MR acquisition sequence, the 3D DSC can be as high as 0.79 (±0.04). These 3D results are also supported by a 2D quantitative evaluation as well as by robustness and variability evaluations. The mean discrepancy (in 2D) between the manual and automatic segmentations for regions around the spinal canal is of 1.8 (±0.8) mm. The robustness study shows that among the five factors evaluated, only the type of MRI acquisition sequence can affect the segmentation results. Finally, the variability of the automatic segmentation method is lower than the variability associated with manual segmentation performed by different physicians.

**Conclusions:**

This comprehensive evaluation of the automatic segmentation and 3D reconstruction of intervertebral disks shows that the proposed technique used with specific MRI acquisition protocol can detect intervertebral disk of scoliotic patient. The newly developed technique is promising for clinical context and can eventually help surgeons during thoracoscopic intervertebral disk resection.

## Background

Depending on the severity of the curve and the risk of progression, scoliosis can be treated with rigorous observation, bracing or surgery. Several types of surgery can be performed on scoliotic patients to reduce their spinal deformations. Precise positioning of hooks and screws on vertebrae attached to a rod, using anterior or posterior approach, to bring back the normal curvature of the spine is an example [1,2]. For patient with stiff curvature, a disk resection is often required to be able to properly attach instrumentations to the rod and optimise the results of the surgery [3,4]. The disk resection is done with a thoracoscope prior to attach hooks or screws to the rod [5]. The removal of the intervertebral disk with only thoracoscopic images is a tedious and challenging task for the surgeon [6]. Intra-operative thoracoscopic images do not fully describe the actual geometry of the structures of interest due to many factors such as the inherent projection of the imaging modality and the small field of view (which leads to loss of depth perception), the presence of surgical tool in the region of interest and other constraints typically imposed in an operating room. The use of computer assistance in such surgeries could help clinicians to perform their surgical manipulations more precisely by dynamically bringing additional information such as the remaining disk tissue or the distance of surgical tools from critical anatomical structures like the aorta or spinal canal. In this context, multimodal image fusion of a preoperative 3D model of the intervertebral disks with intraoperative thoracoscopic images could be very useful to visualize a 3D spine model including soft tissues and bones in a single view, thus reducing cognitive effort on the part of the surgeon.

A variety of imaging modalities can be used to acquire volumetric information on the patient’s anatomy preoperatively. For the current context, MRI is a relevant choice because it is a non invasive imaging modality with the capacity to capture details of soft tissues like intervertebral disks. Manual segmentation of intervertebral disks on every MRI slice is a very time-consuming process and is prone to errors for the radiologist and it is not conceivable in a clinical environment. Time to manually segment one intervertebral disk varies from 15 to 35 minutes depending on the type of MRI sequence, the number of slices covering the volume and the severity of the spinal deformation. Automatic segmentation of intervertebral disks from MRI is an innovative field and it permits reproducible 3D model of the region of interest for an augmented reality system. Towards this goal, we hypothesize that the preoperative 3D geometry of the disk could be extracted from the MRI volume by automatically segmenting the region of interest.

To our knowledge, only one study relates work on segmentation and 3D reconstruction of the intervertebral disk of scoliotic patient [7]. In this study the segmentation of the nucleus pulposus and the annulus fibrosus is done manually. Only few studies relate works on segmentation of MRI spine images from patient with normal spine curvature [8-12]. None of these techniques are useful for our application because of the spinal deformity involved with scoliosis and/or the external constraints namely the unsupervised and closed contours requirements for 3D reconstruction. Indeed, with 3D spine deformation of scoliotic patients, it is impossible to locate the whole spine cord in a single MR image, the intervertebral disks and the verterbrae are often also deformed, bringing an additional challenge to the automatic segmentation method.

Live wire has also been used to segment medical images and it is based on a cost graph principle starting with seeds points given by a user. This technique is a semi automatic segmentation technique that allows the user to select regions of interest by clicking on the images to delineate contour of a specific structure. A graph is built considering pixels as nodes and from each node an edge is created in the four main directions (up,down,left, right). These edges are weighted with features gathered from the sobel filter convolution, so that pixels that stay on the edge are lighter and the ones that go outside the edge are heavier. Several cost function can be used but gradient magnitude is widely used. This type of segmentation is particularly useful to detect complex object contour. Although some work has been done to reduce the problems of the quality of the segmentation and the computational complexity, live wire still necessitates user interaction. Even with the 3D approach [13-16], user has to manually segment the structure of interest on few orthogonal slices or manually delimitate ROI (Region Of Interest) which is a tedious task for the segmentation of more than one intervertebral disk per image.

Watershed has been used in combination with other techniques in cardiology on ultrasound images [17], in neurology on MR images [18,19] and recently we have used this technique on spine MR images [20,21]. The principle of watershed transform is based on the detection of ridges and valleys. The image is viewed as a topological image where intensity represents the altitude of the pixels. The image is flooded from its minimum and it allows the delimitation between the catchment basins and the ridges (watershed lines). Hence the catchment basins represent region of homogeneous intensity representing the region of interest. The results based on the watershed technique showed that the technique is able to cope with variation of topologies and shape and that it was possible to use the algorithm in the sagittal and coronal plane. However, no merging of information (sagittal and coronal plane) was done to encompass the problem of bad boundaries detection in images located at the extreme lateral sides. Also, no 3D quantitative evaluation of this automatic segmentation technique was yet performed.

Segmentation of MR images of patients presenting different degree of scoliotic deformity clearly necessitates the development of a technique able to cope with variations of topology and shape. The purpose of this study is to develop and validate a novel automatic segmentation based on our previous work [20,21]. In these studies only sagittal images or coronal images are used independently. By using only sagittal images, the automatic segmentation had problem to depict interverterbral disk on images located in the lateral side where disks are seen as small structures and are rejected by the automatic segmentation. On the other hand, when the segmentation is done on the coronal images, the same problem occurs on the anterior and posterior side of the intervertebral disk. Hence, using one direction to segment the intervertebral disks on all images covering the volume, in order to have 3D models, is not optimal.

Our first objective is to improve the method developed in [20,21] to take full advantages of MRI by reconstructing coronal images using the sagittal images and merging information from both directions. The second objective is to assess a similarity measure for spatial volumes in order to adequately compare the proposed automatic segmentation results with those of manual segmentation by physicians. Also, because of the 3D spine deformation of scoliotic patients, the intervertebral disks and the verterbrae are often also deformed, bringing an additional challenge to the automatic segmentation method. This clearly motivated the necessity to conduct a robustness evaluation to ascertain the capacity of the proposed technique to cope with different spatial positioning and shape variations of intervertebral disk and surrounding anatomical structures. Hence, the third objective is to evaluate the robustness of the automatic segmentation technique.

## Methods

### Study data

The MR images were acquired at Sainte-Justine Hospital with a 1.5 Tesla Magnetom Avanto system (Siemens, Erlangen, Germany). The radiofrequency (RF) transmitting and receiving units consisted of a body coil. Three different acquisition protocols were studied on 9 scoliotic patients.

The first acquisition protocol was based on a 3D MEDIC (Multi Echo Data Image Combination) sequence used in the sagittal plane with Repetition Time (TR) = 23 milliseconds (ms), Echo Time (TE) = 12 ms, slice thickness of 1 millimeter (mm) and a matrix of 256 X 256 elements leading to a voxel size of 1 mm^3^. The second acquisition used a 3D FISP (Fast Imaging with Steady state Precession) sequence with parameters TR=7.1 ms, TE=2.38 ms, slice thickness of 1 mm and a matrix of 256 X 256 elements leading to a voxel size of 1 mm^3^. The third acquisition protocol was a standard 2D Spin Echo used with parameters TR=780 ms and a TE=18 ms, with a slice thickness of 2 mm and 2.4 mm of space between slices using a matrix of 384 mm X 384 mm, leads to a pixel size of 0.67 mm^2^ in the sagittal direction. The three acquisition protocols were performed in the sagittal plane because our application calls for high resolution in that plane near the spinal canal and intervertebral disks. The three protocols were acquired in the same session, but since these were lengthy acquisitions we allowed the patient to move between each acquisition. The three MRI acquisition sequences were approved by the ethical committee of Sainte-Justine Hospital, Montreal, Canada and a written consent was obtained from the patients or their relative for publication of study.

**Figure 1:**
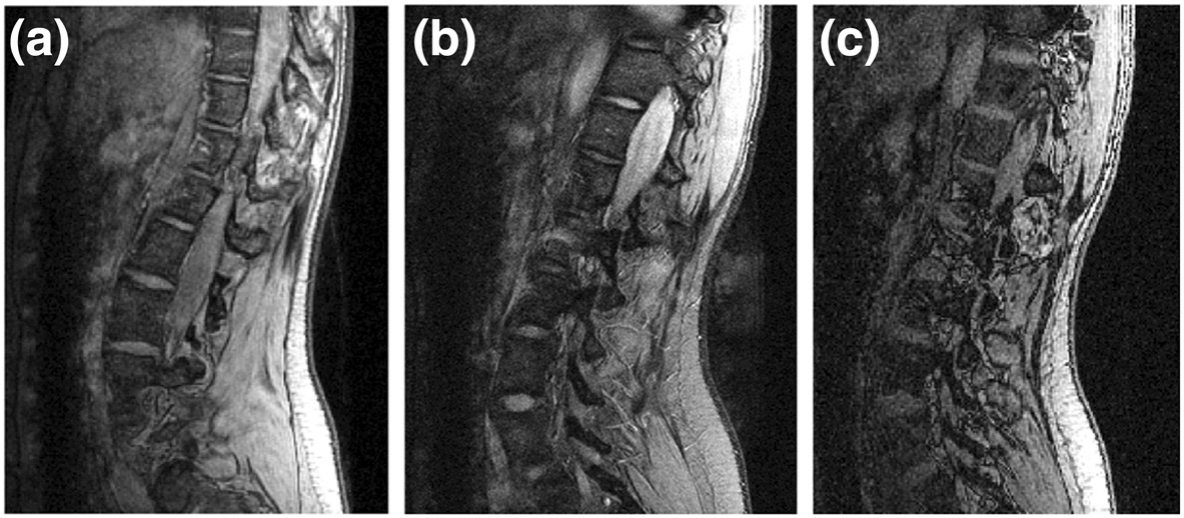
**MRI acquisition protocols.** Three different acquisition sequences for the same patient from the medium scoliotic severity group: (**a**) 3D MEDIC, (**b**) 2D Spin Echo, (**c**) 3D FISP.

The choice of the above acquisition protocols was based on the most commonly used sequence types for segmentation of musculoskeletal images. All three acquisition sequences have relatively short TE’s because this makes it possible to see the intervertebral disks without distinguishing between the annulus and the nucleus pulposus, which is what is required in the current study (Figure 1).

### Automatic reconstruction of intervertebral disks

The proposed algorithm has three main steps: segmentation, classification and fusion of complementary information coming from coronal and sagittal views, thus taking full advantage of the imaging modality. In brief, this algorithm is an unsupervised segmentation technique able to detect intervertebral disks in short TE MR images of scoliotic patients. As a first step, sagittal images and interpolated coronal images are segmented using the watershed technique applied to modified gradient images as reported by our group [20]. The gradient image is modified using internal and external markers and morphological operators to keep only the most significant and relevant contours for the structures of interest. In the context of the watershed method, internal markers (F_int_^m^) represent sets of connected pixels inside the regions of interest, while the external markers (F_ext_^m^) represent the deepest valley lines surrounding every internal marker. Combined binary markers F^m^ were imposed as minima on the gradient image and enabled the automatic segmentation of intervertebral disks in MRI of scoliotic patients. This technique resulted in some over-segmentation, thus necessitating a subsequent classification step.

The classification process, as described in [21], is used exclusively in the sagittal images to label the closed contours as either intervertebral disks or background. In short, the classification step allows us to eliminate background regions that are falsely detected as intervertebral disks in the automatic segmentation step. The supervised k-Nearest Neighbours (k-NN) classifier is used with four statistical and four spectral texture features to label each region as either intervertebral disk or background in the sagittal segmented images. The statistical texture features are based on histogram of the closed contour (mean, standard deviation, skewness and entropy). All four spectral texture features are based on the energy Fourier spectrum of the closed region. By using the Fourier spectrum, we have information about the orientation and the frequency of intensity variation of the closed region. To facilitate interpretation, the spectrum is expressed in polar coordinates (r,Θ). Hence, the 4 descriptors of this function used as spectral texture features are the angle θmax at which the spectrum is maximal, the value S(θ)max of the spectrum at θmax, the variance of S(θ) and the difference between S(θ)max and S(θ)mean [21].

The classification step constitutes the second step of the reconstruction process. Being computationally expensive, the classification is limited to the sagittal images because anatomical correspondence can be performed to locate the intervertebral disk regions in the coronal images.

### Fusion of disk detection in two plane

The third step of the segmentation process is the fusion of information coming from the sagittal and coronal segmentations and it represents the improvement of the technique proposed in [20,21].

**Figure 2:**
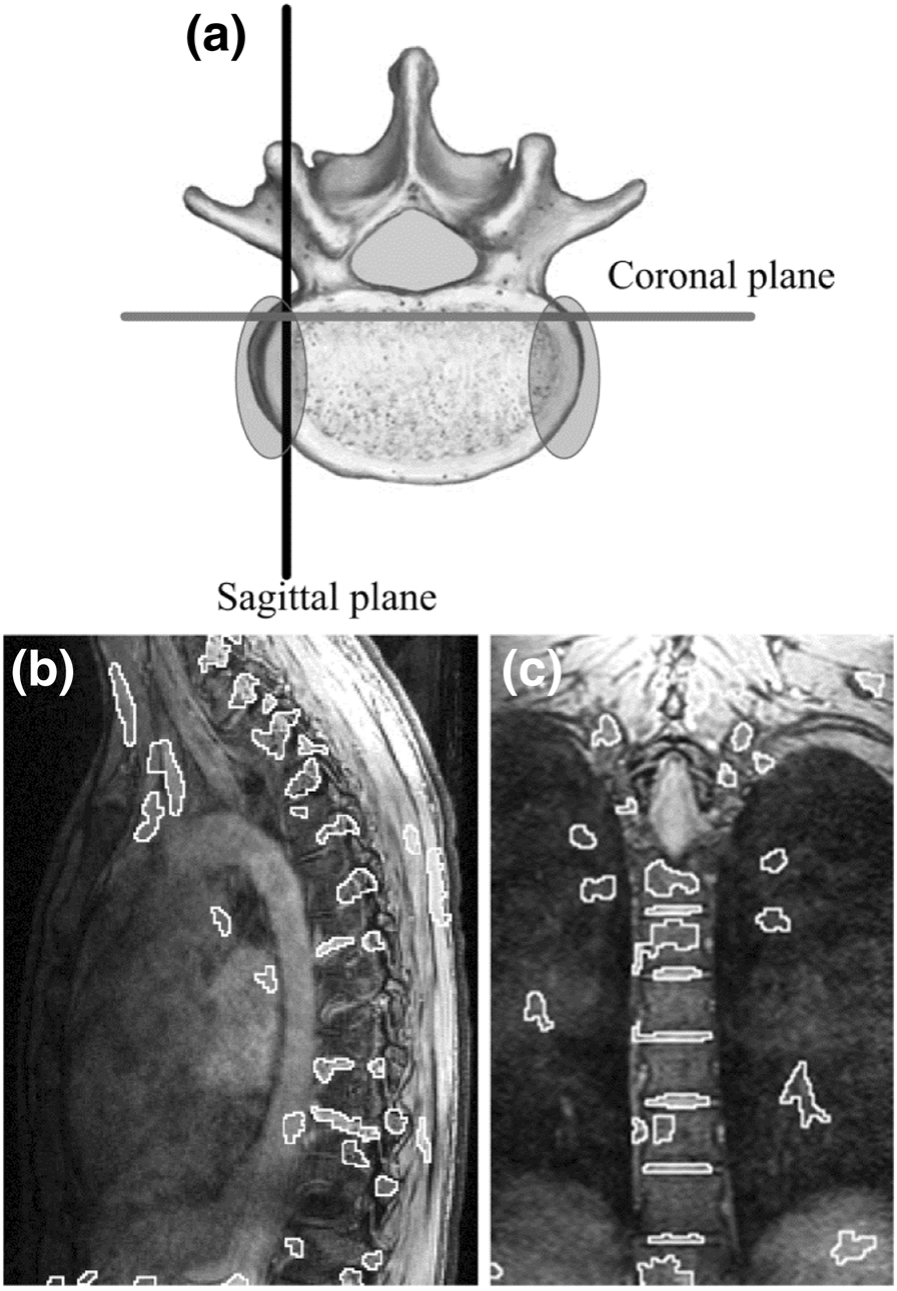
**Problematic zone for automatic segmentation.** Complementary information is found in different imaging planes. (**a**) Axial view of vertebra showing orientations of sagittal and coronal planes. The shaded ellipses show the regions for which intervertebral disks are hard to segment in sagittal images. (**b**) Segmented sagittal image corresponding to the plane showed in (**a**). None of the intervertebral disks are properly segmented in this sagittal image. (**c**) Segmented coronal image corresponding to the plane showed in (**a**). Several disk contours that the automatic segmentation algorithm is not able to detect in the sagittal plane, are on the other hand, well detected by algorithm in the coronal plane.

Hence, merging of information is important because, as illustrated in Figure 2 (b), segmentation of intervertebral disks in sagittal images in the lateral regions of the disk is difficult because of the scoliotic deformity (spinal curvature). But, regions where the disks are hard to identify in the sagittal plane, corresponds to regions where disks can easily be segmented in the coronal plane (Figure 2 (c)). The inclusion of the reconstructed coronal image segmentation data thus helped us to delimit more precisely the lateral portions of the disks, corresponding to the shaded elliptical areas in Figure 2 (a). The implementation of this third step addresses the problem of missing sections of the lateral part of the disks as it was pointed out in previous study [20].

**Figure 3:**
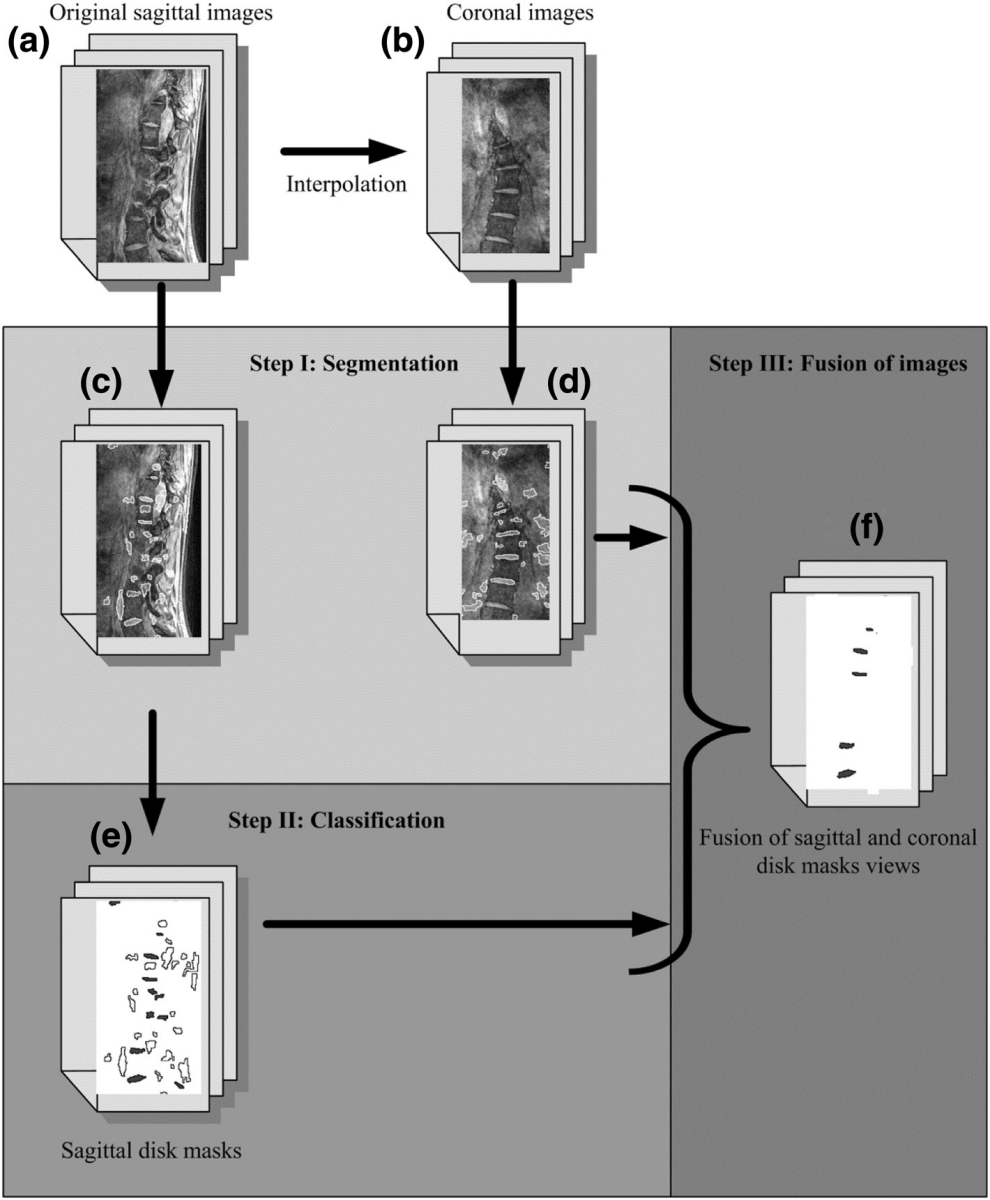
**Flowchart of the novel automatic segmentation process.** Flow chart of the different steps necessary to obtain the final reconstruction of the intervertebral disks.

Coronal images are reconstructed from the sagittal images without the need of adding another direction of image acquisition during the MRI protocol. From these newly created coronal images, we apply the same segmentation process as for the sagittal images. The fusion of sagittal and coronal segmentation information is achieved as follows: because the slice thickness and the spacing between slices were known, it is possible to join the voxels of the sagittal disk masks and create a reference volume for each disk. The same process is applied to the unlabelled segmented coronal images, creating a set of volumes representing disks and background regions in that case. The centroids of the volumes are calculated and represented the key points for anatomical correspondence between the volumes created from the classified regions in the sagittal plane (reference disk volumes) and the set of volumes created from the segmentation of the coronal planes. Corresponding disk volumes from the two imaging planes are thus superimposed using a union operator. The fusion of the two volumes representing the same disk *D* is based on the following equation:

(1) D=Dref∪Dcor

where *Dref* is the disk volume coming from the sagittal segmentation and *Dcor* is the disk volume coming from the segmentation of coronal images directly. The union of the coronal segmentation with sagittal segmentaion allows us to fill empty spaces (created by missing disk detection) encounters in the lateral portion of the disk if only sagittal images where used (Figure 2). Moreover, this step enables us to use all the information provided by a volume acquisition modality like MRI and addresses the difficulty of disk segmentation in the lateral regions of the disks when analyzing sagittal images only [21]. A summary of the three steps of the reconstruction process is shown in Figure 3.

### Evaluation of the automatic segmentation

To conduct a quantitative evaluation of the automatic segmentation, comparison with gold standard is necessary. The manual segmentations of intervertebral disks performed by 3 clinical experts were considered the gold standard. The validation dataset consists of nine scoliotic patients who underwent magnetic resonance imaging with the three different protocols. Three of the patients have a mild main thoracic curve (Cobb angles from 12° to 24°), three have a moderate curve (Cobb angles from 28° to 35°) and three have a more severe curve requiring surgery (Cobb angles from 43° to 60°). Each user has segmented a total of nine intervertebral disks coming from different patients presenting different curve severities and representing different MRI protocols and different positions relative to the apex of the curve. Experts have carefully indicated the boundaries of the intervertebral disks in every MR slice using the commercially available SliceOmatic™ software (Tomovision, Montreal). While doing the manual segmentation, the experts had access to the orthogonal views, just as in the proposed automatic segmentation approach.

### Similarity measure

The Dice Similarity Coefficient (DSC) is used as a statistical metric to evaluate the performance of the novel automatic segmentation method. The DSC has been used in various studies to evaluate the segmentation of many organs in MRI and CT [22-25]. The DSC measures the spatial overlap between two segmentations X and Y. The coefficient is defined as:

(2) $$DSC(X,Y)=\frac{2\left|X\cap Y\right|}{\left|X\right|+\left|Y\right|}$$

where X represents the set of voxels in an intervertebral disk resulting from automatic segmentation and Y the set of voxels contained in the same intervertebral disk resulting from manual segmentation. The values for the DSC range between 0 and 1 where 0 means no overlap and 1 means a perfect overlap between the manual segmentation performed by one of the experts and the corresponding automatic segmentation. A DSC value greater than 0.7 has been reported as indicating good segmentation performance [22,23,25].

The number of voxels contained in the volumes produced by manual and automatic segmentation is also compared to provide an indicator of over- or under-estimation of volumes created by the automatic method.

To refine the evaluation of the automatic segmentation, the 2D DSC is also calculated for every sagittal image to locate the intervertebral disk regions that are more prone to produce large errors using automatic segmentation. Moreover, a mean 2D distance in mm between the manually segmented and automatic segmented disk boundaries in the area of the spinal canal is calculated in the sagittal images spanning the canal.

### Variability

The variance of the results is calculated to compare the variability of the DSC for the proposed segmentation algorithm with the inter-user variability. The inter-user variability represents the degree of concordance between manual segmentations performed by different users. To evaluate the variability, the same three disks are segmented by two clinical experts for each MRI acquisition protocol. The 3D DSC is calculated using (Eq.1) for three different cases: a first case for which manual segmentation of user 1 is compared with the automatic segmentation results, a second case for which manual segmentation of user 2 is compared with the automatic segmentation results and a third case for which manual segmentation of user 1 is compared with manual segmentation of user 2.

### Robustness

As the second objective of the paper is to evaluate the robustness of the automatic segmentation algorithm, we have evaluated whether the results of the automatic segmentation are influenced by specific factors characterizing the MR images of scoliotic patients. An experimental factorial design is used to determine the effect of five major characteristics of MR images of scoliotic patients. The studied factors are: 1) the type of MRI acquisition sequence, 2) the position of the intervertebral disk relative to the apex of the curvature, 3) the degree of severity of the curvature, 4) the MRI inter-patient variability for each image acquisition protocol, and finally 5) the user who performed the manual segmentation.

The Table 1 was automatically created with STATISTICA™ from StatSoft Inc. (Oklahoma, U.S.) and it summarizes the setting of the modalities for each factor and each segmentation. Each factor has three modalities represented by 1, 0 and −1 in Table 1. The modalities for the MRI acquisition sequence type are the three acquisition sequences described earlier (3D MEDIC, 3D FISP and 2D Spin Echo). The modalities for the severity of the scoliosis are low severity, moderate severity and high severity deformation. For the position of the intervertebral disk relative to the apex, the modalities corresponds to the disk located at the apex itself, one level superior to the apex and one level inferior to the apex. Three different users have performed manual segmentation and represent the three user modalities. Finally, to verify if the MRI inter-patient variability for a given MRI acquisition sequence can modify the result of the DSC measure, the manual segmentation is divided into three blocks, each block representing a trio of patients with low, moderate and high severity deformations. This is how uncontrollable factor like MRI inter-patient variability can be taken into account in this type of robustness study.

**Table 1:** Overview of the modality settings for each segmentation created by Statistica

**Run**	**Block**	**Cobb**	**Position**	**MRI**	**User**
1	1	1	0	−1	−1
2	1	0	1	1	0
3	1	−1	−1	1	0
4	1	0	1	−1	−1
5	1	1	0	1	0
6	1	−1	−1	0	1
7	1	1	0	0	1
8	1	−1	−1	−1	−1
9	1	0	1	0	1
10	2	−1	0	0	0
11	2	−1	0	−1	−1
12	2	1	1	1	−1
13	2	0	−1	−1	1
14	2	0	−1	0	0
15	2	0	−1	1	−1
16	2	1	1	0	0
17	2	−1	0	1	−1
18	2	1	1	−1	1
19	3	0	0	1	1
20	3	0	0	−1	0
21	3	−1	1	0	−1
22	3	0	0	0	−1
23	3	1	−1	1	−1
24	3	1	−1	−1	0
25	3	1	−1	0	−1
26	3	−1	1	1	1
27	3	−1	1	−1	0

With a factorial design taking into account simple and double interactions of four factors, and with three blocks of patients to also consider the uncontrollable factor related to MRI inter-patient variability, the number of runs is 27: 3^(4–2)^ x 3 blocks = 27, or *M*^*(k-p)*^ where *M* is the modality, *k* the number of factors and *p* the number of higher order interactions that the user wants to eliminate. With this type of statistical evaluation, it is possible to evaluate whether the simple and double interactions of the factors have an effect on the studied response and also whether the blocking factor has an effect on the studied response with a minimal number of runs. The ANOVA analysis and Pareto chart of effects will allow us to verify which factor or combination of factors affect the results. This part of the evaluation is performed using STATISTICA™ from StatSoft Inc. (Oklahoma, U.S.).

## Results

### 3D similarity measure

The study included segmentation of 27 intervertebral disks coming from 9 scoliotic patients and 3 different MRI acquisition protocols. Table 2 shows the 3D DSC values and their standard deviation for the three MRI sequences and the number (*n*) of disks used for the evaluation. Within a group for a given MRI sequence, each of the *n* disks comes from a different patient. An ANOVA test shows that the 3D DSC of the 3D FISP protocol is statistically lower than the results obtained with the other two protocols. However there is no statistically significant difference between the results obtained for the 3D MEDIC and the Spin Echo sequences. The mean value of the 3D DSC for those two sequences is 0.77 and is higher than the threshold value of 0.70, considered as the minimum for a good segmentation performance.

**Table 2:** **Mean 3D Dice Similarity Coefficient and its standard deviation for the three acquisition sequences, each group composed of*n*disks**

**MRI Sequences**	**3D DSC (std dev)**	**n**
3D MEDIC	0.79(0.04)	9
3D FISP	0.64(0.09)	9
Spin Echo	0.75(0.07)	9
All sequences	0.73(0.09)	27
3D MEDIC and Spin Echo	0.77(0.06)	18

**Figure 4:**
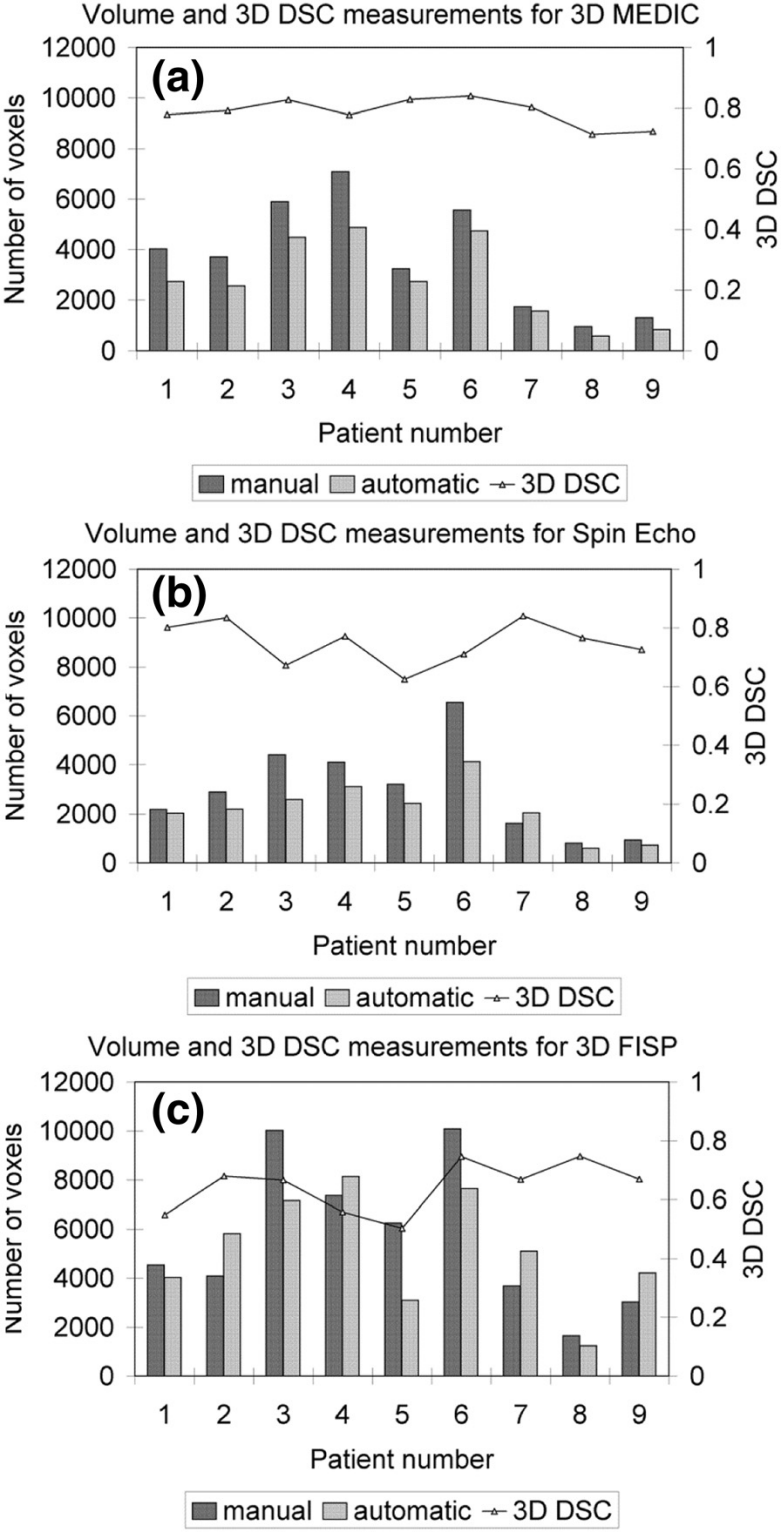
**Volume and 3D DSC measurements.** The three graphs represent the volumes of the 27 intervertebral disks obtained manually and automatically and the 3D DSC values for each MRI acquisition sequence: (**a**) for the 3D MEDIC, (**b**) for the Spin Echo and (**c**) for the 3D FISP.

To assess the segmentation performance in terms of over- or under-segmentation of the volume of an intervertebral disk, the number of voxels is calculated for the 27 different intervertebral disks. For each type of MRI sequence, there are a total of nine disks, each corresponding to a different patient, and representing different Cobb angles and different positions relative to the apex. Figure 4 shows that for the 3D MEDIC (a) and Spin Echo (b), the majority (all except one) of the automatic segmentations were underestimated compared to manual segmentation (by 25% and 20% respectively). For the 3D FISP (Figure 4 (c)), there is no trend in the over- or under-segmentation of volume for the automatic segmentation compared to the manual segmentation. For this MRI sequence, the automatic volume is either under- or over-estimated by mean 30% compared to manual segmentation.

**Figure 5:**
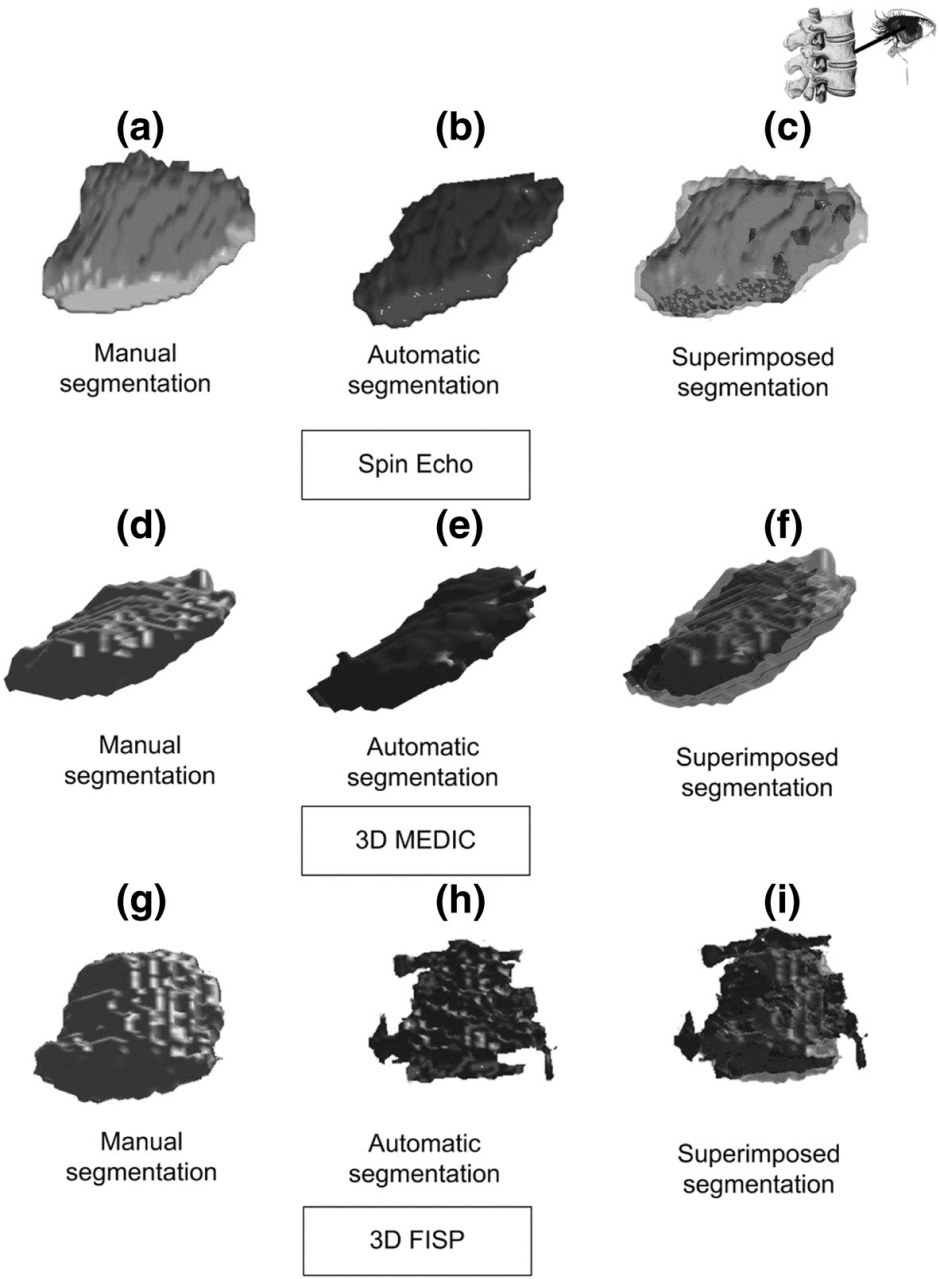
**3D reconstruction of intervertebral disks.** 3D reconstruction of intervertebral disks obtained with the manual and automatic segmentation procedures. The first column shows a 3D reconstruction in light gray representing the manual segmentation; the second column shows a 3D reconstruction in dark gray representing the automatic segmentation. The third column shows the superimposition of the manual and automatic segmentations. The first row shows the results for the Spin Echo MRI sequence, the second row shows the results for the 3D MEDIC and the third row shows the results for the 3D FISP.

Typical results for the 3D reconstruction of intervertebral disks are shown in Figure 5 for the three different MRI acquisitions. By superimposing the volumes and applying transparency (Figure 5 (c) and (f)), it is clear that for the 3D MEDIC and Spin Echo sequences, the error in the spatial overlap between the manual and automatic volumes, as calculated with the 3D DSC, is mainly due to volume underestimation by the automatic process. On the other hand, for the 3D FISP, we can see that the 3D reconstruction of the automatic segmentation (Figure 5 (h)) has more visual discontinuity in the contour than the results obtained for the two other MRI acquisition protocols (Figure 5 (b) and (e)) and than the corresponding manual result (Figure 5 (g)).

### 2D similarity measure

**Figure 6:**
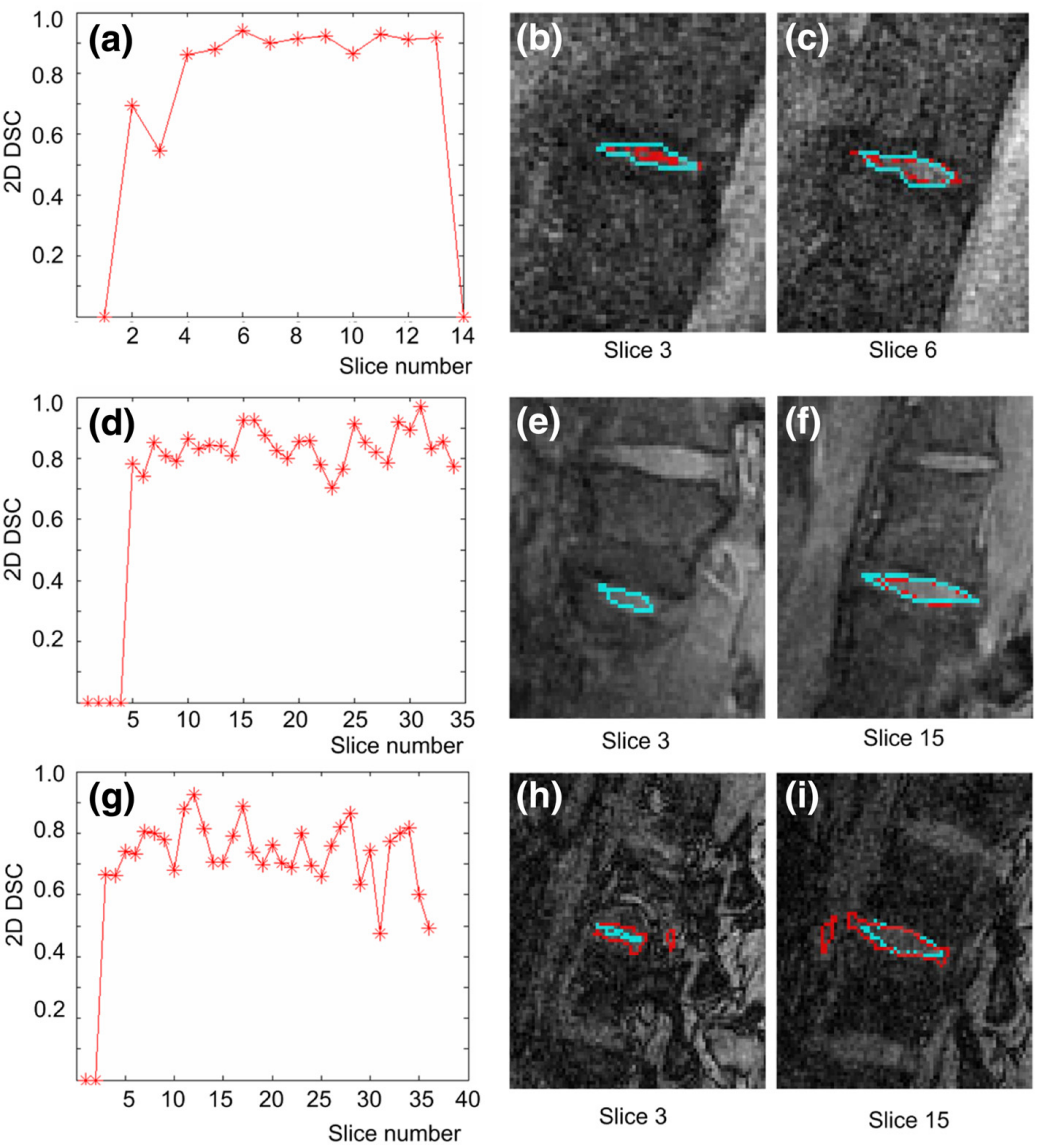
**2D measurements.** Graphs of the 2D DSC relative to slice number are presented for the (**a**) Spin Echo, (**d**) 3D MEDIC and (**g**) 3D FISP sequences. In the 2D images, cyan represents manual segmentation and red represents automatic segmentation of the intervertebral disk for slices in the lateral and mid-sagittal planes of the disk for the Spin Echo ((**b**) and (**c**)), 3D MEDIC ((**e**) and (**f**)), and 3D FISP (**h**) and (**i**) sequences.

To complete the performance evaluation of the novel automatic segmentation algorithm, the 2D DSC is calculated on all slices of all the intervertebral disk volumes in this study. Tables 3, 4 and 5 present the mean 2D DSC for the mid-sagittal slices and for the lateral slices for the 3D MEDIC, Spin Echo and 3D FISP MRI sequences respectively. The mid-sagittal slices correspond to 80% of the slices spanning the intervertebral disks, while the remaining 20% are the lateral slices (10% on each side). The number of slices composing each intervertebral disk is also indicated (*n*). Examination of the results shows that the 2D DSC values for the mid-sagittal slices are always higher than for the lateral slices for every given volume in the case of the 3D MEDIC and Spin Echo images, while this is not the case for the 3D FISP images. Figure 6 shows in detail some typical results for the 2D DSC.

**Table 3:** Mean 2D DSC and standard deviation for the mid-sagittal and lateral slices for 3D MEDIC images of nine patients

**3D MEDIC Patients**	**Mid-sag slices Mean DSC**	**std dev**	**n**	**Lateral slices Mean DSC**	**std dev**	**n**
p1	0.70	0.29	28	0.52	0.48	8
p2	0.79	0.14	30	0.06	0.12	8
p3	0.86	0.06	32	0.36	0.31	8
p4	0.78	0.17	40	0.05	0.16	10
p5	0.83	0.06	26	0.43	0.46	8
p6	0.83	0.11	33	0.40	0.38	10
p7	0.81	0.09	22	0.65	0.31	6
p8	0.73	0.09	23	0.13	0.24	6
p9	0.66	0.30	25	0.31	0.43	8

**Table 4:** Mean 2D DSC and standard deviation for the mid-sagittal and lateral slices for Spin Echo images of nine patients

**3D MEDIC Patients**	**Mid-sag slices Mean DSC**	**std dev**	**n**	**Lateral slices Mean DSC**	**std dev**	**n**
p1	0.82	0.09	9	0.58	0.25	4
p2	0.83	0.04	9	0.82	0.10	4
p3	0.69	0.08	13	0.26	0.30	4
p4	0.74	0.25	12	0.67	0.21	4
p5	0.56	0.33	11	0.36	0.30	4
p6	0.64	0.30	14	0.58	0.14	4
p7	0.86	0.12	9	0.63	0.43	4
p8	0.74	0.32	8	0.29	0.41	2
p9	0.67	0.28	9	0.28	0.42	4

**Table 5:** Mean 2D DSC and standard deviation for the mid-sagittal and lateral slices for 3D FISP images of nine patients

**3D MEDIC Patients**	**Mid-sag slices Mean DSC**	**std dev**	**n**	**Lateral slices Mean DSC**	**std dev**	**n**
p1	0.43	0.35	29	0.42	0.36	8
p2	0.75	0.09	28	0.50	0.33	8
p3	0.58	0.30	31	0.38	0.41	10
p4	0.41	0.18	36	0.44	0.09	10
p5	0.35	0.34	32	0.11	0.19	10
p6	0.63	0.27	32	0.70	0.16	8
p7	0.66	0.12	26	0.45	0.36	8
p8	0.74	0.22	28	0.20	0.28	8
p9	0.66	0.07	24	0.61	0.27	8

Figure 6 (a), (d) and (g) plot the distribution of the 2D DSC as a function of slice position. These graphs demonstrate that the lower DSC values are found in the lateral slices for the 3D MEDIC and Spin Echo. It is also possible to appreciate visually the under-segmentation by the automatic method in the case of the 3D MEDIC and Spin Echo sequences, which occurs mainly in the lateral slices (lateral portions of the disk) as can be seen in Figure 6 (b) and (e). On the other hand, the mid-sagittal slices (Figure 6 (c) and (f)) show very similar contours for the manual and automatic segmentations. For the 3D FISP case however, the 2D contours show no systematic under-segmentation of the intervertebral disk contours (Figure 6 (h) and (i)) and significant variations of the 2D DSC values are found throughout the volume.

We also sought to quantify the discrepancy between the 2D boundaries of the manual and automatic segmentations in the sagittal imaging slices surrounding the spinal canal, for the Spin Echo and 3D MEDIC sequences. In this evaluation, we find that the automatic segmentation was underestimated (*i.e.* its boundary was farther from the edge of the canal) compared to the manual segmentation by an average distance of 3.4 mm (±1.5mm) and 1.8 mm (±0.8mm) for those two sequences respectively.

### Variability of the 3D DSC results

When developing segmentation algorithms for clinical imaging data, an accepted gold standard is the corresponding manual segmentation, but the latter introduces inter-user variability. In our case, a comparison of the variance of the results of the proposed segmentation algorithm with the variance of the manual segmentation results for two users shows that for the 3D MEDIC and Spin Echo sequences, the variability introduced by the automatic segmentation is lower than the inter-user variability for both users. Table 6 is based on a total of 9 intervertebral disks segmented twice by 2 clinical experts.

**Table 6:** Mean 3D DSC with standard deviation comparing automatic segmentation against manual segmentation performed by users 1 and 2 and comparing manual segmentation of user 1 against manual segmentation of user 2

**Compared volumes**	**3D MEDIC 3D DSC (std dev)**	**Spin Echo 3D DSC (std dev)**	**3D FISP 3D DSC (std dev)**	**n**
User 1 vs automatic	0.77 (0.05)	0.70 (0.05)	0.59 (0.07)	3
User 2 vs automatic	0.72 (0.05)	0.76 (0.04)	0.60 (0.10)	3
User 1 vs User 2	0.81 (0.07)	0.78 (0.05)	0.72 (0.07)	3

The number of disks is specified by n.

Table 6 shows that the standard deviations for the inter-user results were 0.07 and 0.05 for the 3D MEDIC and Spin Echo respectively. These values are higher than the variability obtained with the proposed automatic segmentation process. For the 3D FISP, the automatic segmentation leads to a variability of 0.10 when compared to user 2. This variability is higher than the inter-user variability of 0.07. Also for the 3D FISP, when comparing user 1 against automatic segmentation, the variability is the same as the inter-user variability.

### Robustness

**Figure 7:**
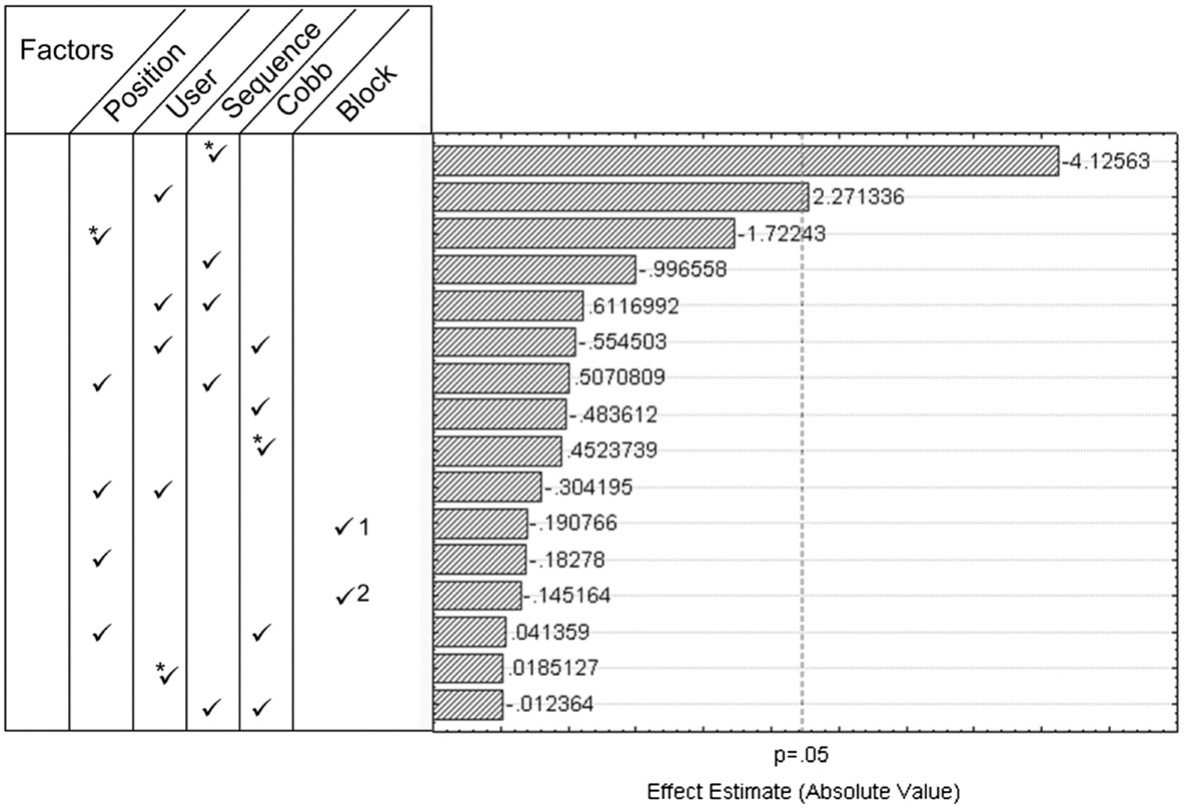
**Pareto chart.** Pareto Chart of the estimated effects of the 4 controllable factors and 1 uncontrollable factor (Block) on the DSC values. The four controllable factors are the position of the disk relative to the apex of the curvature, the user performing the manual segmentation, the MRI acquisition sequence and the Cobb angle. The uncontrollable factor is the block corresponding to the inter-patient variability. Block 1, Block 2 and Block 3 illustrate the three groups used to create the block. The asterisk * represents the quadratic term and if not specified, the linear term is considered in the equation for the statistical model used to estimate the effect. If more than one factor is specified, this means that the interaction of the 2 specified factors is studied.

The ANOVA analysis and Pareto chart of effects (Figure 7) reveal that the type of MRI sequence is by far (p = 0.000285) the factor that most affects the prediction equation. The user also affects the values of the DSC, but to a lower degree (p = 0.0418) than the MRI acquisition sequence. The inter-patient MRI intensity variability (block), the severity of the scoliosis (Cobb angle) and the position of the studied intervertebral disk relative to the apex of the spinal deformity do not influence the response of the system as reported by the 3D DSC measurement. Results also show that interactions between the different factors do not affect the response of the system.

## Discussion

Using clinical dataset from real scoliotic patients is important for this study because topology of the spine on MR images varies a lot from normal to scoliotic patient adding important challenge in the segmentation process. In this context, it is commonly accepted to set manual segmentation as gold standard. Aside from the Dice Similarity Coefficient (DSC) used to test the validation criteria, the calculation of volume is also part of the evaluation. The similarity coefficient has the advantage of taking into account the spatial dependency, which is not the case when reporting volumes only. Conversely, although geometrically intuitive, the DSC lacks information about the type of segmentation error, namely whether over- or under-segmentation occurs. By taking into account both metrics (DSC and volume), the current study provides a comprehensive quantitative evaluation of the automatic segmentation applied to a clinical dataset composed of 27 intervertebral disks coming from nine scoliotic patients.

From the comparison of the automatic segmentation with manual segmentation, we find that the proposed algorithm yields to spatial volumes that are similar to the gold standard, since the average 3D DSC values of 0.79 for the 3D MEDIC and 0.75 for the Spin Echo (Table 2) are higher than the 0.7 threshold for good segmentation performance.

No other study on segmentation based on region detection for 3D reconstruction of intervertebral disk of scoliotic patient exists. However Michopoulou *et al [*[12]. have an automatic segmentation for intervertebral disk based on a priori shape information and fuzzy c-mean algorithm. Their segmentation procedure is applied on the mid-sagittal image to evaluate if the disk is degenerated. They have evaluated their segmentation accuracy using a 2D DSC value on the mid-sagittal image. Hence we can partly compare our results with this study. On Figure 6 of the current study, the 2D DSC value for the Spin Echo at the mid-sagittal image (image 7) is 0.9 and 0.88 for the 3D MEDIC at the mid-sagittal level. This is comparable to the results obtained by Michopoulou. Indeed they obtained 0.88 (for the elastic-Atlas-RFCM method), 0.84 (for the Atlas-FCM method) and 0.87 (for the Atlas-RFCM method) on degenerated disk.

Results reveals that the reconstructed 3D volumes of intervertebral disks are systematically underestimated (mean discrepancy of 22.5%) compared to volumes obtained with manual segmentation performed on 3D MEDIC and Spin Echo MR images. For the 3D FISP, there is no trend in over- or under-segmentation but there is a mean discrepancy of 30% between the automatic volumes and the manual volumes. Indeed, there is less consistency from slice to slice for the 3D FISP images because the automatic segmentation algorithm has trouble with the blurred boundaries of intervertebral disks often found in the 3D FISP sequences. The three clinical experts who performed the manual segmentation all agreed that the intervertebral disks were harder to delimitate in the 3D FISP sequences because of the blurred contours (due to variation of pixel intensities along the boundaries). Hence, even in the manually identified volumes, there is less consistency from slice to slice compared to the two other types of MR images.

The volume underestimation resulting from the automatic segmentation algorithm applied to 3D MEDIC and Spin Echo images occurs more in the lateral slices than in the mid-sagittal slices. Superimposition of volumes in space and 2D evaluation of the DSC (see Tables 3 and 4) show higher 2D DSC results in the mid-sagittal slices than in the lateral slices for all patients, meaning that the differences between the volumes lie mainly in the lateral regions of the disks. For the 3D FISP (Table 5), there is no specific region of volume under- or over-estimation since the results for the 2D DSC vary as much in the mid-sagittal slices as in the lateral slices.

For surgeons, the underestimation of the volume of anatomical structures is viewed as a margin of safety in a computer assistance system. Indeed, by reasonably underestimating the working volume (e.g. the intervertebral disk), surgeons will have more confidence in the 3D model, since they will know that if their surgical tools are inside the 3D model there is no chance to injure critical anatomical structures (e.g. the spinal cord). For example, in spinal release before instrumentation of scoliotic patient, the intervertebral disk must be partially removed and delicate anatomical structures surrounding the disk like the spinal canal and aorta must not be injured during the procedure. These structures are located to the anterior left side of the disk (for the aorta) and to the posterior side (for the spinal canal). The distance in mm between the manual and the automatic segmentations in the sagittal slices spanning the spinal canal is of 3.4 mm (±1.5mm) for the Spin Echo and 1.8 mm (±0.8mm) for the 3D MEDIC. The greater underestimation of the disk for the Spin Echo sequence can be explained by the fact that for half of the patients, the Spin Echo sequence resulted in images with some pixels being brighter in the nucleus compared to the annulus, thus misleading the automatic segmentation process which detected the nucleus boundary as the external disk boundary. A modification of the parameters of the Spin Echo sequence would eliminate this discrepancy between the results in mm of the 3D MEDIC and Spin Echo sequences. Hence, for a disk resection application, a mean underestimation distance of 1.8 mm in the mid-sagittal planes compared to manually segmented contours gives an adequate margin of safety.

The variability associated with the use of automatic segmentation is lower than the variability associated with manual segmentation performed by different users. This is true for both the 3D MEDIC and Spin Echo MR sequences, therefore making the use of the automatic segmentation method clinically feasible. Hence, this study addresses an important issue concerning the use of computer assistance in a clinical environment. Indeed, for an automatic segmentation algorithm to be acceptable, the variability of the 3D model on which the computer assistance system relies should be equal to or lower than the variability of an equivalent 3D model obtained from manual segmentation.

One of the limits of the study is that for the three MRI sequences, the Field Of View (FOV) encompasses only five to seven vertebral levels. It is well known that scoliotic patients often have double curvature (one in the thoracic region and one in the lumbar region of the spine). With such a small FOV it is not possible to image both curvatures at a time. This limitation also entails that in the robustness evaluation, the effect of the position of the disk relative to the spinal region (thoracic or lumbar) has not been considered. Because thoracic disks are smaller than lumbar disks, the behavior of the automatic segmentation algorithm may vary for different vertebral levels. In the current study, the spinal curves included in the MRI were mainly in the lumbar and lower thoracic regions.

However, the robustness study does include an evaluation of the effects of five important factors. Results show that the proposed automatic segmentation algorithm is robust, in light of the fact that the results for the 3D DSC are not affected by the severity of the spinal deformity, the position of the disk relative to the apex or the inter-patient MR intensity variation. On the other hand, the type of MR acquisition sequence is important and could substantially affect the results of the automatic segmentation. Considering that the mean 3D DSC value is significantly lower for the 3D FISP than for the other two sequences (Table 2), the 3D FISP MR acquisition protocol is not recommended for good performance of the proposed automatic segmentation method.

The recommended MR acquisition protocols for the proposed intervertebral disk automatic segmentation method are thus the Spin Echo and 3D MEDIC MR sequences. There is no statistical difference between the 3D DSC results of for these two protocols. The choice between 3D MEDIC and Spin Echo will depend on the clinician. The acquisition time of the 3D MEDIC sequence is 2.5 times longer than for Spin Echo. A longer acquisition time means less reproducible results because patients are more prone to move during the acquisition. Depending on the clinician and on the application, one might decide to use Spin Echo even if some interpolation is required between slices in order to reconstruct in 3D, because an acquisition time of only 12 minutes is more feasible and will have more chance of giving non-blurred images for all patients.

## Conclusions

Although the applicability of this method is limited to specific MRI acquisition protocol, the proposed automatic segmentation of intervertebral disks on scoliotic patient is accurate, reliable, and reproducible tool for volume extraction of intervertebral disk of scoliotic patient. The proposed automatic segmentation algorithm is able to cope with patients presenting varying degrees of scoliotic spinal deformity. This work is an important step toward providing reliable pre-operative model updated using intra-operative images to help surgeons to visualize structures of interests during surgeries such as disk resection.

## Competing interests

The authors declare that they have no competing interests.

## Authors' contributions

CC and FC made substantial conceptual contributions to the design of the study, analysis and interpretation of data, and contributed to drafting of the manuscript. GG and M-CM were involved in manual segmentation and in image data analysis and interpretation of the MRI, and gave their input from a clinical point of view. C-EA gave critical revision of the manuscript regarding important intellectual content. All authors have read and approved the final version of the manuscript.

## Pre-publication history

The pre-publication history for this paper can be accessed here:

http://www.biomedcentral.com/1471-2342/12/26/prepub
